# Navigating Cultural Diversity in the Selection of Cardiovascular Device Treatments: A Comprehensive Review

**DOI:** 10.7759/cureus.38934

**Published:** 2023-05-12

**Authors:** Md. Rockyb Hasan, Tahsin Tabassum, Tanzin Tabassum, Mohammed A Tanbir, Mahzabin Kibria, Mahidul Chowduary, Rajesh Nambiar

**Affiliations:** 1 Department of Internal Medicine, Amarillo Campus, Texas Tech University Health Sciences Center, Amarillo, USA; 2 Department of Public Health, School of Community Health and Policy, Morgan State University, Baltimore, USA; 3 Department of General Surgery, West Suffolk Hospital, Bury St Edmunds, GBR; 4 Department of Medicine, Sir Salimullah Medical College, Dhaka, BGD; 5 Department of Internal Medicine, Interfaith Medical Center, Brooklyn, USA; 6 Department of Cardiology, Amarillo Campus, Texas Tech University Health Sciences Center, Amarillo, USA

**Keywords:** devices in cardiology, socioeconomic barriers in cardiology, religious barriers in cardiology, language barriers in cardiology, cultural barriers in cardiology

## Abstract

In cardiology, patients' cultural beliefs, linguistic differences, lack of knowledge, and socioeconomic status can create barriers to choosing device treatment. To address this issue, we conducted a thorough literature review using online databases such as PubMed, Google Scholar, and Texas Tech University Health Sciences Center's research portal. Our review found that cultural, religious, and linguistic barriers can contribute to patients' apprehension and reservations about device placement. These barriers can also impact patients' adherence to treatment and clinical outcomes. Patients from lower socioeconomic backgrounds may have difficulty accessing and affording device-based treatments. Additionally, fear and inadequate understanding of surgical procedures can deter patients from accepting device treatment in cardiology. To overcome these cultural barriers, healthcare providers must raise awareness about the benefits of device treatment and provide better training to overcome these challenges. It is crucial to address the unique needs of patients from different cultural backgrounds and socioeconomic statuses to ensure they receive the care they need.

## Introduction and background

Miss X, a 60-year-old African American woman, was admitted to the hospital with cardiac chest pain and was diagnosed with non-ST-elevated myocardial infarction. Her medical history revealed hypertension and type 2 diabetes. Following a coronary angiogram, the cardiology team recommended coronary artery bypass graft surgery, which Miss X declined, opting for alternative treatment options. After careful evaluation, the cardiology team suggested the use of a medical device, along with standard treatment, to manage her condition. They recommended an implantable cardiac defibrillator (ICD), which could monitor her heart rhythm and prevent sudden cardiac arrest from dangerous arrhythmias. Despite a detailed discussion of the benefits of this device, Miss X refused treatment. She cited cultural and religious reasons for her refusal. She believed that the placement of foreign objects in her body would violate her personal beliefs and cultural practices. The medical team sought the assistance of a cultural liaison to understand her perspective and identify alternative treatment options that were culturally sensitive. The medical team and Miss X agreed to try noninvasive therapies such as medication, diet, and lifestyle changes to manage her cardiac condition. She was advised to regularly follow up and provided ongoing support for her adherence to a noninvasive treatment plan. The case of Miss X highlights the importance of cultural and religious factors in healthcare decision-making, particularly for patients of diverse origins. The medical team’s efforts to understand Miss X’s perspective and find alternative treatment options that respected her cultural beliefs were crucial in achieving a positive outcome for her cardiac health.

In the field of cardiology, technology plays a critical role in diagnosing and treating various heart conditions. As technology continues to advance, the number of devices available for use in cardiology continues to increase. Over the last two decades, the advancement of cardiology has introduced various medical devices, including pacemakers, ICDs, and cardiac resynchronization therapy (CRT) devices to the clinical arena [1]. These devices have shown both morbidity and mortality benefits [1]. However, cultural barriers can affect the choices made by healthcare professionals and patients when it comes to selecting devices.

One of the main cultural barriers affecting device selection in cardiology is the belief system of healthcare professionals and patients [2]. Different cultures have different beliefs about health and illness, which can influence their choice of medical treatments and devices. For example, some cultures may prefer natural remedies and traditional healing methods over modern medical devices. In contrast, other cultures may place a strong emphasis on technology and prefer the latest devices, even if they are not necessary. Another cultural barrier is language. In many cases, healthcare professionals and patients come from different cultural and linguistic backgrounds. This can make it challenging to communicate effectively about medical devices and their uses. Misunderstandings can occur, leading to inappropriate or ineffective device selection. Healthcare professionals in cardiology need to be aware of these language barriers and take steps to overcome them, such as using interpreters or translated materials [3]. Cultural beliefs and values can also impact the level of trust healthcare professionals and patients have in certain devices. In some cultures, there may be a lack of trust in modern medical devices due to a history of mistreatment or experimentation. For example, some African American patients may be reluctant to use certain heart devices due to a history of unethical medical experimentation on Black patients in the United States [4-5]. This lack of trust can lead to reluctance to use certain devices, even if they have shown benefits in clinical trials. 

Additionally, cultural barriers can impact the availability and accessibility of certain devices in different regions of the world. In some developing countries, for example, there may be limited access to advanced medical technology due to economic and infrastructural limitations. This can lead to a preference for more basic devices or alternative therapies that may be more readily available. Similarly, in some cultures, there may be a preference for locally made devices over imported ones, even if the imported devices are of higher quality. The consequences of cultural barriers to device selection in cardiology can be significant. In some cases, inappropriate device selection can lead to ineffective treatment or even harm the patient. For example, a patient may be given a device that they are not comfortable with or do not trust, leading to noncompliance and poorer outcomes [6]. In other cases, limited access to advanced medical technology can result in a lack of access to lifesaving treatments [7-8]. To overcome the cultural barriers in device selection in cardiology, healthcare professionals need to be aware of the cultural beliefs and values of their patients and be sensitive to their needs. This may involve providing information in a culturally appropriate manner or using interpreters or translated materials to ensure effective communication. Additionally, healthcare professionals should work to build trust with their patients and address any concerns they may have about medical devices. This may involve acknowledging historical injustices or explaining the benefits and risks of different devices. 

Cultural barriers can significantly influence the selection of devices in cardiology. Healthcare professionals must be aware of these barriers and take steps to overcome them to ensure effective treatment and positive outcomes for their patients. By understanding the cultural beliefs and values of their patients, healthcare professionals can select the most appropriate devices and build trust with their patients, ultimately leading to better healthcare outcomes.

## Review

We identified the first research topic as *Cultural Barriers in Choosing Cardiologic Devices*. This topic was broken down further into the following specific research questions: What cultural factors influence the selection of cardiology devices in different regions of the world? Or how do cultural beliefs and practices affect patient and physician preference for cardiology devices? Once the research question was identified, we conducted a comprehensive search of the existing literature on the topic. We used a variety of sources, such as scholarly journals, academic databases, and books. PubMed, Google Scholar, and Texas Tech University Health Sciences Center’s research portal are the online databases that were used for developing this review. A combination of key terms such as cultural, linguistic, religious, and socioeconomic barriers in cardiology and devices in cardiology was used to retrieve relevant, peer-reviewed articles for analysis from these databases. Upon listing the key terms in the selected databases' search engines, 71,528 hits were found. We looked for studies that have explored cultural barriers to the adoption of cardiology devices, as well as studies that have investigated the role of cultural factors in medical decision-making more broadly. We analyzed 156 selected articles to identify key themes and trends related to cultural barriers in cardiology device selection. We used a thematic analysis approach to identify patterns across the literature. After examining the literature, we synthesized the information to create a cohesive narrative that outlines the major cultural barriers to cardiology device selection. We identified key factors contributing to these barriers such as religious beliefs, socioeconomic status, and cultural norms. We discuss the implications and recommendations below. We considered recommendations for improving cross-cultural communication and understanding between physicians and patients, as well as strategies for addressing cultural barriers in cardiology device selection.

Patients who do not speak the same language as their healthcare provider may have difficulty understanding their diagnosis, management options, type of device offered by the clinician, and post-procedure instructions. Language and communication barriers often lead to improper device placement and poor adherence to medication and lifestyle changes, which will ultimately affect patient outcomes [9-10]. Evidence shows that linguistic barriers affect decision-making, faith, and the involvement of clergy, communicating with family about complications, prognosis, and follow-up [11].

Religious beliefs often present barriers to certain medical treatments as well as the choice of devices in cardiology. For example, some Jehovah's Witnesses refuse a blood transfusion, even in a life-threatening situation, based on their religious beliefs [12-13]. This can present challenges for cardiology patients who may require transfusions during surgery and to treat the bleeding disorder. Additionally, these beliefs can pose a challenge in the management of associated bleeding with device implantation or anticoagulation therapy. For example, heparins are often derived from pigs and some Muslim and Jewish patients may object to taking medications derived from pigs, as these animals are considered prohibited in their respective religions [14-15]. Many religions prescribe periods of fasting, during which food and drink are restricted [16]. Following a device placement or after a cardiac stent, certain medications are required to be taken with food or at certain times of the day. In these cases, adhering to their treatment regimen during the period of fasting is a challenging situation. Orthodox Jews may observe strict dietary laws that prohibit the consumption of certain foods, including pork and shellfish [17]. This can affect the use of some anticoagulant medications, such as heparin. Alternative medications, such as enoxaparin, which is derived from nonporcine sources, may need to be considered in these cases. Sometimes, some Christians believe in the power of prayer and spiritual healing, and as such, they may refuse medical treatment, including device implantation [18]. In cases where device treatment is medically necessary, healthcare providers may need to work with the patient and their family to address their concerns and ensure that they receive appropriate care.

Native Americans typically have a holistic view of health and sickness. They believe in a delicate balance between nature, spirituality, people, and the greater community. Imbalance disruption can lead to illness and/or death as per their popular belief system [19]. In Chinese, Korean, and Vietnamese cultures, illness and death are viewed as a natural part of life, and they often refuse device placement in cardiology [20]. Oftentimes, Vietnamese families describe the illness as a conflict between the body and nature, and device placement conversation is challenging. Furthermore, Buddhist, Confucian, or Hindu faiths may attribute the concept of bad karma to illness and suffering and view suffering as a mechanism for atoning for sins committed in the former life [21-22]. Their acceptance of fate in cardiac disease often challenges initiating conversations about device placement. Korean and Russian people sometimes view disease as arising from interfamilial or peer conflict. Latinos often interpret death as something both natural and uncontrollable and believe that fate is often left in the hands of God. Some African cultures believe that pacemakers are unnatural and that the heart should be left to beat on its own [23]. Thus, different belief systems often play an important role in choosing device placement in cardiology. In traditional Chinese medicine, treatment often includes various body and mind exercises alongside herbal medicine. Device treatment often is a new choice for patients of Chinese descent, which will need a holistic discussion. Additionally, family plays a part in each member's health. In Western medicine, health is a private issue that is closely guarded, sometimes even from friends and family. In Asian cultures, there is a strong communal aspect of health [24]. Three generations live in the same household, and health is openly discussed [24]. Thus, individuals of Asian descent often rely on extensive family discussions before choosing device therapy in cardiology. Muslim patients may have concerns about using devices, especially Arab-Muslim patients' religious concerns also suggest the need for cultural competence and sensitivity on the part of healthcare practitioners [25].

One ethnographic study *Evidence-Based Cross-Cultural Health Knowledge of the Montagnard Vietnamese Community* in Greensboro, North Carolina, showed patterns found among Montagnard refugees, including misunderstandings between the patient and the provider due to differences in defining illness, passive obedience, reliance on traditional medicine, and false perceptions of Western medicine [26]. 

Oftentimes, patients may not understand the benefits of device treatment or how it works, leading to reluctance to undergo treatment. Some patients may be afraid of undergoing surgery to implant the device, especially if they have never had surgery before. Certain alternative therapies, such as acupuncture and electrical stimulation, may interfere with the function of implanted devices, such as pacemakers and ICDs. Sometimes, alternative medicine therapies often lack the scientific evidence to support their effectiveness in treating heart conditions [27]. As a result, patients may opt for alternative therapies instead of device placement, which can be more effective. Patients may receive misinformation about device treatment or alternative therapies, leading to distrust of both types of treatment.

Socioeconomic status can be a barrier to cardiac device treatment in cardiology, as lower income patients may face challenges accessing and affording these treatments. Patients with lower socioeconomic status may have limited access to ICDs due to the high cost of devices and procedures. A study published in *JAMA Cardiology* in 2020 found that patients from lower socioeconomic status patients are less likely to receive an ICD, even after accounting for clinical factors and insurance coverage [28]. Lower socioeconomic status has difficulty accessing CRT due to factors such as lack of compliance with medical appointments, lack of health insurance, and limited availability of specialized cardiac centers. A study published in the *Journal of Cardiac Failure* in 2019 found that patients with low-socioeconomic status were less likely to receive CRT than those with higher socioeconomic status. Left-ventricular-assisted devices (LVADs) are oftentimes expensive, and many patients do not have access to specialized LVAD centers [29]. A study published in the *Journal of Cardiac Failure* in 2016 found that lower income patients were less likely to receive LVAD and had higher rates of mortality compared to higher income patients [29].

The lack of diversity in clinical trials is another significant cultural obstacle that can impede the adoption of medical devices among minority communities. While clinical trials are essential in establishing the safety and effectiveness of medical devices, a lack of diversity in trial participants can restrict the generalizability of the results. This can cause skepticism among minority communities toward devices that have not been tested on people who resemble them. A study found a notable gap in the use of prophylactic aspirin for preventing coronary artery disease between whites (34.7%) and African Americans (27.2%) with a statistically significant difference (*P *< 0.0001) [30]. A cross-sectional analysis conducted between 2006 and 2011 revealed that individuals who received an ICD were significantly more likely to be white (58.8%) than African American (11%). The analysis also indicated that most American ICD recipients during this period were white [31]. In comparison to white patients, black and Hispanic patients who were eligible for CRT were less likely to receive the therapy. According to a multivariate analysis, black patients (odds ratio [OR] 0.84; 95% confidence interval [CI], 0.75-0.95; *P* < 0.004) and Hispanic patients (OR 0.83; 95% CI, 0.71-0.99; *P* < 0.033) who were eligible for CRT were found to be less likely to receive the treatment [32]. This disparity underscores the adverse consequences of limited diversity in cardiology clinical trials, including device treatment. The primary factor contributing to this inequality is the persistence of institutional racism and biases within the healthcare system, which diminishes trust among many minority communities and reduces their participation in clinical trials [33]. Additionally, social and economic factors such as inadequate access to healthcare, education, and income can also negatively impact the involvement of underrepresented groups in cardiology clinical trials. These issues can cause African American individuals to be more hesitant than their white counterparts when it comes to selecting cardiac devices due to a lack of representation and diversity in clinical trials, leading to a perception that the devices may not have been tested adequately on people who look like them.

## Conclusions

Cultural barriers are a crucial factor that can impact the adoption of cardiology devices. These barriers can arise from differences in cultural beliefs, values, and attitudes toward healthcare, medical technology, and the role of physicians. Cultural barriers can affect the acceptance and utilization of cardiac devices, leading to disparities in the quality of care and patient outcomes. Communication between patients and physicians is essential for effective treatment, but language barriers can make it difficult for patients to understand the benefits and risks of cardiac devices. In addition, cultural beliefs about illness and treatment can influence patients' willingness to accept cardiac devices. Cultural differences in healthcare systems can also affect the adoption of cardiac devices. In some cultures, the role of the physician is highly respected, and patients may not question their recommendations. In other cultures, patients may be more assertive and want to be more involved in their healthcare decisions. Cardiology device manufacturers must understand these cultural differences to develop products that meet the needs and expectations of different patient populations. To address cultural barriers to the adoption of cardiac devices, there must be a concerted effort to increase diversity in clinical trials. This can help to ensure that devices are safe and effective for all patient populations. In addition, healthcare providers must be trained to be culturally sensitive and aware of the different cultural beliefs and practices that may affect patients' willingness to accept cardiac devices. Patient education and outreach can also play a crucial role in overcoming cultural barriers. Patients must be educated on the benefits and risks of cardiac devices in a culturally sensitive and relevant manner. This can include using language and communication methods that resonate with patients from different cultures. Collaboration between healthcare providers and cardiology device manufacturers is critical in developing products that meet the needs and expectations of different cultural groups. Manufacturers must engage with patients and healthcare providers to understand their cultural beliefs, preferences, and attitudes toward cardiac devices. This can help develop products that are more acceptable and accessible to patients from different cultures. Cultural barriers to the adoption of cardiac devices are complex and multifactorial. Addressing these barriers requires a concerted effort from all stakeholders, including patients, healthcare providers, and device manufacturers. By understanding and addressing these cultural barriers, we can ensure that all patients have access to safe, effective, and culturally appropriate cardiac devices.
